# Eco-Friendly Methodology to Prepare *N*-Heterocycles Related to Dihydropyridines: Microwave-Assisted Synthesis of Alkyl 4-Arylsubstituted-6-chloro-5-formyl-2-methyl-1,4-dihydropyridine-3-carboxylate and 4-Arylsubstituted-4,7-dihydrofuro[3,4-*b*]pyridine-2,5(1*H*,3*H*)-dione

**DOI:** 10.3390/molecules16119620

**Published:** 2011-11-21

**Authors:** Hortensia Rodríguez, Osnieski Martin, Margarita Suarez, Nazario Martín, Fernando Albericio

**Affiliations:** 1 Laboratory of Organic Chemistry, Department of Organic Chemistry, Chemistry Faculty, University of Havana, 10400, Cuba; Email: osniesky@gmail.com (O.M.); msuarez@fq.uh.cu (M.S.); 2 Institute for Research in Biomedicine, Barcelona Science Park, Baldiri Reixac 10, 08028 Barcelona, Spain; 3 Department of Organic Chemistry, Chemistry Faculty, Universidad Complutense, 28040 Madrid, Spain; Email: nazmar@quim.ucm.es (N.M.); 4 CIBER-BBN, Networking Centre on Bioengineering, Biomaterials and Nanomedicine, Barcelona Science Park, Baldiri Reixac 10, 08028 Barcelona, Spain; 5 Department of Organic Chemistry, University of Barcelona, Martí i Franqués 1-11, 08028 Barcelona, Spain

**Keywords:** microwave, dihydropyridines, green chemistry

## Abstract

Here we describe the efficient synthesis of alkyl 4-arylsubstituted-6-chloro-5-formyl-2-methyl-1,4-dihydropyridine-3-carboxylates and 4-arylsubstituted-4,7-dihydro-furo[3,4-*b*]pyridine-2,5(1*H*,3*H*)-diones via microwave-accelerated reaction of alkyl 4-arylsubstituted-2-methyl-6-oxo-1,4,5,6-tetrahydro-3-pyridinecarboxylates with the appropriate reagents. This eco-friendly approach to these valuable dihydropyridine derivatives does not involve the harsh or highly contaminating conditions common in classical heating and offers a reduction or even elimination of solvent use and recovery, simplification of the work-up procedures, facility of scale up, and low energy consumption, in addition to moderate to higher yields.

## 1. Introduction

Nitrogen heterocycles are frequently found in privileged (pharmacophore) structures [1,2], but their incorporation is often hindered (multistep sequences, lack of generality, preparation from acyclic precursors, *etc.*); thus, only a limited number of strategies have been successfully applied in the synthesis of heterocyclic scaffolds [3,4,5]. The development of new, rapid, and clean synthetic routes toward focused libraries of such compounds is of relevance to medicinal and synthetic chemists alike [6]. Undoubtedly, the most efficient strategies involve multicomponent reactions (MCRs), which are powerful tools for the rapid introduction of molecular diversity [7,8]. Consequently, interest in the design and development of MCRs for the generation of heterocycles is growing [9].

In recent years, increasing interest has been focused on the synthesis of 1,4-dihydropyridine derivatives (1,4-DHPs) owing to their significant biological activity [10,11,12]. In particular, dihydropyridine drugs, such as nifedipine, nicardipine, amlodipine, and others, are effective cardiovascular agents for the treatment of hypertension [13]. However, in spite of the potential utility of these drugs, their synthesis usually involves expensive reagents, organic solvents, long reaction times, and affords unsatisfactory yields. Thus, the development of an efficient and versatile method for the execution of of the Hantzsch reaction is an active ongoing field of research, and there is scope for further improvements in the form of milder reaction conditions, shorter reaction times, and improved yields [14,15,16].

The application of microwave irradiation (MW) as a non-conventional energy source for the activation of reactions, in general and under solvent-free conditions in particular, has now gained popularity compared to standard homogeneous and heterogeneous reactions because it provides enhanced reaction rates and (usually) improved product yields. In addition, in the context of green chemistry, MW irradiation has several eco-friendly advantages, which have been extended to modern drug discovery processes. Generally, the rapid heating induced by MW avoids the harsh classical conditions and decomposition of reagents, thus facilitating the formation of products under milder reaction conditions with a consequent increase in yield [17,18,19].

**Figure 1:**
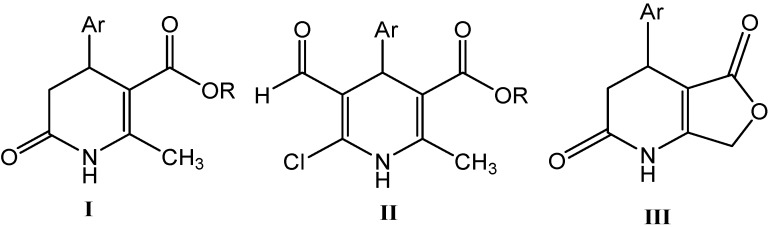
Chemical structures of alkyl 4-arylsubstituted-2-methyl-6-oxo-1,4,5,6-tetrahydro-3-pyridinecarboxylates **I**, alkyl 4-arylsubstituted-6-chloro-5-formyl-2-methyl-1,4-dihydropyridine-3-carboxylates **II** and 4-arylsubstituted-4,7-dihydrofuro[3,4-*b*]pyridine-2,5(1H,3H)-diones **III**.

In the context of our general interest in MCRs and as part of our ongoing research programs into non-conventional synthesis as an eco-friendly approach to produce nitrogen heterocyclic compounds, here we report the MW-assisted synthesis (MWAS) of alkyl 4-arylsubstituted-6-chloro-5-formyl-2-methyl-1,4-dihydropyridine-3-carboxylates **II** and 4-arylsubstituted-4,7-dihydrofuro[3,4-*b*]pyridine-2,5(1*H*,3*H*)-diones **III** from alkyl 4-arylsubstituted-2-methyl-6-oxo-1,4,5,6-tetrahydro-3-pyridine-carboxylates **I**. The 1,4-DHPs bearing the chlorine and formyl group proved to be useful intermediates for the synthesis of other pyridine-fused heterocycles (Figure 1).

## 2. Results and Discussion

To obtain the alkyl 4-arylsubstituted-6-chloro-5-formyl-2-methyl-1,4-dihydropyridine-3-carboxylates **IIa–j** and 4-arylsubstituted-4,7-dihydrofuro[3,4-b]pyridine-2,5(1H,3H)-dione DHP derivatives **III**, we previously synthesized alkyl 4-arylsubstituted-2-methyl-6-oxo-1,4,5,6-tetrahydro-3-pyridinecarboxylates **Ia–j**. Briefly, compounds **Ia–j** were prepared under solvent-free conditions in a one-pot condensation reaction assisted by MW irradiation using the methods previously reported by our group [20,21]. When the irradiation was stopped, the solids were treated with appropriate solvents and filtered to give the pure products **Ia–j** in moderate to good yields (44%–89%).

For the MWAS, the irradiation was provided by a CEM Discover LabMate Focused Single Mode MW Synthesis System, which allows for continuous stirring and irradiation with temperature control [22]. All the reactions were followed by TLC and the experiments were replicated in order to ensure reproducibility.

### 2.1. Microwave Assisted Synthesis (MWAS) of Methyl 4-Arylsubstituted-6-chloro-5-formyl-2-methyl-1,4-dihydropyridine-3-carboxylates ***IIa–j***

Some 6-chloro-5-formyl-1,4-dihydropyridine derivatives have been prepared by reaction of alkyl 2-methyl 6-oxo-1,4,5,6-tetrahydropyridine-3-carboxylates **I** with Vilsmeier-Haack reagent (POCl_3_, DMF) [12,23,24,25]; however, these reactions require long times (18 h) to obtain moderate or good yields. We recently reported on the ultrasound-assisted synthesis of these derivatives and found considerable improvements over conventional Vilsmeier-Haack chloroformylation [26]. In addition, MW irradiation has been used to accelerate the Vilsmeier-Haack formylations of pyrrole substrates [27].

**Scheme 1:**
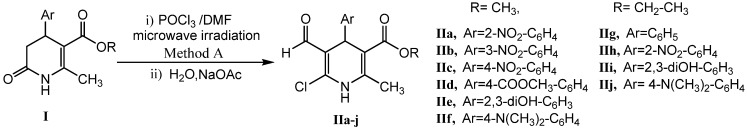
Synthesis of compounds **IIa–j**.

The MWAS of compounds **IIa–j** was performed in a one-step procedure by reaction in an open vessel with previously prepared POCl_3_/DMF. The MW-accelerated Vilsmeier-Haack reaction is typically carried out in a MW reactor at 180 Watts and a controlled temperature of 50 °C for 5 min. Subsequent hydrolysis produces almost analytically pure compounds **IIa–j**, which requires minimal if any purification (Scheme 1).

Table 1 shows the results obtained for the MWAS of compounds **IIa–j** and the comparison with the classic method previously reported by our group (Method B) [24,25].

**Table 1:** Results of MWAS (Method A) of alkyl 4-arylsubstituted-6-chloro-5-formyl-2-methyl-1,4-dihydropyridine-3-carboxylate (**IIa–j**) and comparison with the conventional method (Method B) [24,25].

Product	R	Ar	Method	T(°C)	t (min)	Yield (%)
**IIa**	CH_3_	2-NO_2_-C_6_H_4_	A	50	5	69
			B	RT	1080	70
**IIb**	CH_3_	3-NO_2_-C_6_H_4_	A	50	5	68
			B	RT	1080	73
**IIc**	CH_3_	4-NO_2_-C_6_H_4_	A	50	5	65
			B	RT	1080	69
**IId**	CH_3_	4-COOCH_3_-C_6_H_4_	A	50	5	63
			B	RT	1080	73
**IIe**	CH_3_	2,3-diOH-C_6_H_4_	A	50	5	70
			B	RT	1080	63
**IIf**	CH_3_	4- *N*(CH_3_)_2_-C_6_H_4_	A	50	5	60
			B	RT	1080	68
**IIg**	CH_2_CH_3_	C_6_H_5_	A	50	5	62
			B	RT	1080	75
**IIh**	CH_2_CH_3_	2-NO_2_-C_6_H_4_	A	50	5	65
			B	RT	1080	75
**IIi**	CH_2_CH_3_	2,3-diOH-C_6_H_4_	A	50	5	63
			B	RT	1080	71
**IIj**	CH_2_CH_3_	4- *N*(CH_3_)2-C_6_H_4_	A	70	5	64
			B	RT	1080	70

In all cases, the yields for these compounds by MWAS (Method A) were slightly lower than those previously reported for conventional synthesis [24,25], although the reaction times were dramatically reduced from 18 h under conventional synthesis to 5 min for MWAS. With MW irradiation as an energy source, the partial decomposition of the Vilsmeier-Haack (VH) reagent was promoted by the increase in reaction temperature, taking to account the presence of *N,N-*dimethylformamide in the reaction mixtures. This polar molecule is highly sensitive to MW irradiation and allows higher temperatures [28,29]. In fact, under our MW-assisted chloroformylation conditions, the presence of DMF masked the specific effect of MWs, and the accelerations are attributed mainly to the superheating effect of the solvent. However, in order to determine whether there is a specific MW effect accelerating the reaction with respect to conventional heating, we carried out all the experiments using classical heating (thermostated oil bath) in the same conditions as under MWAS (time, profiles of rise in temperature, vessels, *etc.*). When the starting materials were heated at 50 °C for 5 min to 2 h without solvent, only a complex mixture of by-products was detected (TLC and ^1^H-NMR).

**Scheme 2:**
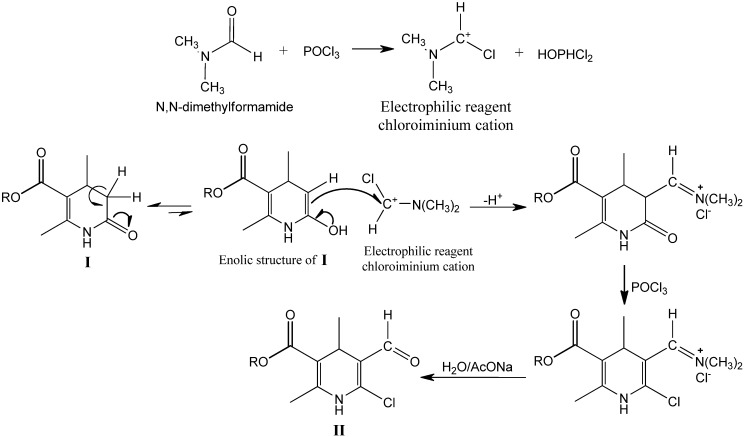
The proposed mechanism of the chloroformylation reaction.

The improvement achieved with MWAS (Method A) could be associated with the reaction mechanism and the evolution of polarity during the MW-assisted reaction. This reaction was shown to proceed through a mechanism involving several steps, beginning with the formation of the electrophilic VH reagent from DMF and POCl_3_. Reaction of the electrophilic reagent with the enolic form of the intermediate compound **I** proceeded through a pyridone intermediate, followed by reaction with POCl_3_ to give the chloro derivative intermediate, which, via subsequent hydrolysis, provided the desired 6-chloro-5-formyl-1,4-DHP **II** (Scheme 2).

**Scheme 3:**
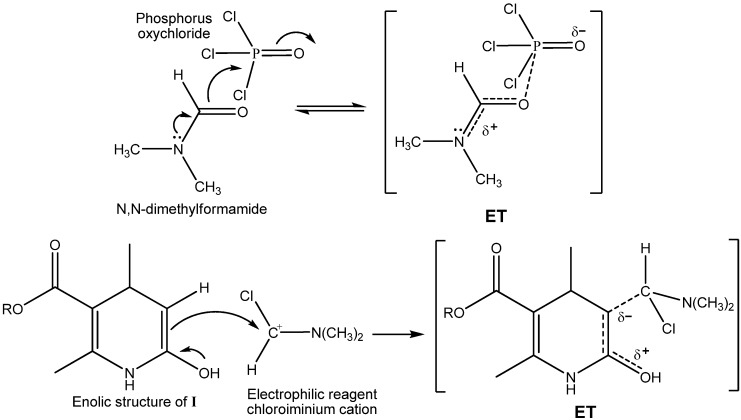
Postulated transition states (**ET**) for the formation of VH reagent and for the reaction between the electrophilic reagent and the enolic form in the chloroformylation reaction.

On the basis of the previous result and the postulated mechanism, we propose that the specific MW effect is attributable to the following: Improvements in the formation of the chloroiminium species (VH reagent), and the enhancement of the subsequent reaction of the electrophilic reagent with the enolic form of the intermediate compound **I**. In both cases, the polarity increased from the ground state to the transition state (Scheme 3), thereby resulting in an enhancement of reactivity as a result of a decrease in the corresponding activation energy [30].

The final products **IIa–j** were characterized by melting point, NMR and mass spectral data. Most compounds synthetized in this study were known and their spectral characterization showed satisfactory agreement with previous literature data [12,23,24,25]. The ^1^H-NMR spectra of the DHP derivatives **IIa–j** showed two singlets at δ ~ 10.6 ppm and δ ~ 9.6 ppm, corresponding to the NH and CHO protons, respectively. The singlet corresponding to the H4 proton appeared in the range of δ 4.9–5.3 ppm and the methyl group on C-2 as a singlet at δ ~ 2.3 ppm. The alkoxycarbonyl group on C3 appeared as a singlet (δ ~ 3.5 ppm) in the case of R = CH_3_ (compounds **5a–f**) and as a quadruplet-triplet when R = CH_2_CH_3_ (compounds **5g–j**) at δ ~ 3.9 ppm and δ ~ 1.1 ppm, respectively. The ^1^H-NMR spectra also showed signals corresponding to the phenyl protons, depending upon the substitution present on the aromatic ring. The ^13^C-NMR spectra of these compounds displayed signals in the carbonyl, aromatic and aliphatic regions. For the nitrogen heterocyclic ring, the spectra showed four quaternary carbon signals (C-2, C-3, C-5, and C-6), and one secondary carbon signal (C-4). The formyl group (CHO) carbon in these systems appeared at 187–186 ppm. The alkoxycarbonyl group appeared at 166.2–166.9 ppm.

MWAS of chloroformyl derivatives **IIa–j** offers considerable improvements over our previously reported conventional Vilsmeier-Haack chloroformylation [12,23,24,25]. The reaction time was notably reduced (conventional synthesis: 18 h, and MWAS: 5 min), and the final product was obtained with excellent purity and hence could be used in further synthetic steps without any need for wasteful purification.

### 2.2. Microwave Assisted Synthesis (MWAS) of 4-Arylsubstituted-4,7-dihydrofuro[3,4-b]pyridine-2,5(1H,3H)-diones ***IIIa–i***

**Scheme 4:**
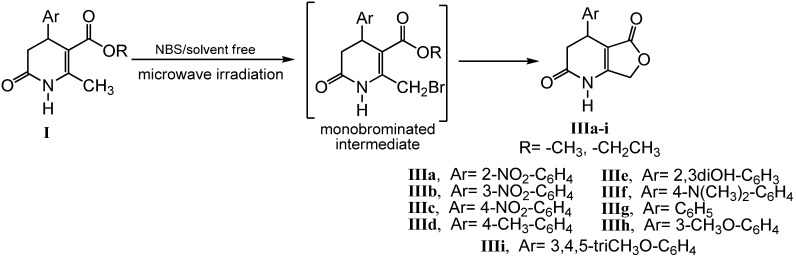
Schematic representation for the synthesis of compounds **IIIa–i**.

Some substituents on the 1,4-DHP ring have a dramatic effect on its biological activities [31]. Specifically, cyclohexanone and γ-lactone rings fused to the 1,4-DHP moiety result in a striking effect on the entry of calcium ions into the intracellular space (calcium antagonist effect) [32]. Our classical method for the synthesis of 4-arylsubstituted-4,7-dihydrofuro[3,4-b]pyridine-2,5(1H,3H)-diones **III** in moderate to good yields, comprised a one-pot reaction of the alkyl 2-methyl 6-oxo-1,4,5,6-tetrahydropyridine-3-carboxylates **I** with *N*-bromosuccinimide (NBS) as the brominating reagent by refluxing in chloroform in 12–14 h [33,34,35]. The microwave-accelerated lactonization to obtain the furo[3,4-b]pyridines **IIIa–i** were performed in a one-step procedure by reaction of previously synthesized **I** with NBS without solvent. This reaction was carried out at 240 Watts and under a controlled temperature of 80 °C for 10 min (Scheme 4). When the irradiation was stopped, the mixture was treated with the adequate solvents and filtered to give the pure products **IIIa–i** in excellent yields.

Table 2 shows the results obtained for the MWAS of compounds **IIIa–i**, compared with the classical method previously reported by our group (Method B) [33,34,35]. To check the possibility of intervention of specific non-pure thermal effects of MWs, the reaction was performed by heating in thermostated oil bath under the same experimental conditions used for MW irradiation (time, profiles of rise in temperature, vessels). In no case was a reaction detected by TLC at 10 min of reaction, and after 2 h of reaction the TLC showed a complex mixture of byproducts.

**Table 2:** Results of MWASwithout solvent (Method A) of 4-arylsubstituted-4,7-dihydro-furo[3,4-b]pyridine-2,5(1H,3H)-diones **IIIa–j** and comparison with the conventional method (Method B) [33,34,35].

Product	R	Ar	Method	T(°C)	t (min)	Yield (%)
**IIIa**	-CH_3_	2-NO_2_-C_6_H_4_	**A**	80	10	80
	-CH_2_CH_3_			80	10	85
	-CH_3_		**B**	62	720	55
	-CH_2_CH_3_			62	720	60
**IIIb**	-CH_3_	3-NO_2_-C_6_H_4_	**A**	80	10	79
	-CH_2_CH_3_			80	10	82
	-CH_3_		**B**	62	720	52
	-CH_2_CH_3_			62	720	58
**IIIc**	-CH_3_	4-NO_2_-C_6_H_4_	**A**	80	10	82
	-CH_2_CH_3_			80	10	85
	-CH_3_		**B**	62	720	55
	-CH_2_CH_3_			62	720	57
**IIId**	-CH_3_	4-CH_3_-C_6_H_4_	**A**	80	10	89
	-CH_2_CH_3_			80	10	85
	-CH_3_		**B**	62	720	61
	-CH_2_CH_3_			62	720	56
**IIIe**	-CH_3_	2,3-diOH-C_6_H_4_	**A**	80	10	72
	-CH_2_CH_3_			80	10	79
	-CH_3_		**B**	62	720	52
	-CH_2_CH_3_			62	720	55
**IIIf**	-CH_3_	4-N(CH_3_)_2_-C_6_H_4_	**A**	80	10	84
	-CH_2_CH_3_			80	10	85
	-CH_3_		**B**	62	720	59
	-CH_2_CH_3_			62	720	53
**IIIg**	-CH_3_	C_6_H_5_	**A**	80	10	84
	-CH_2_CH_3_			80	10	85
	-CH_3_		**B**	62	720	59
	-CH_2_CH_3_			62	720	61
**IIIh**	-CH_3_	3-CH_3_O-C_6_H_4_	**A**	80	10	88
	-CH_2_CH_3_			80	10	85
	-CH_3_		**B**	62	720	51
	-CH_2_CH_3_			62	720	58
**IIIi**	-CH_3_	3,4,5-triCH_3_O-C_6_H_4_	**A**	80	10	83
	-CH_2_CH_3_			80	10	81
	-CH_3_		**B**	62	720	63
	-CH_2_CH_3_			62	720	59

In all cases, the MWAS yields for these compounds (Method A) were higher than those achieved previously with conventional synthesis conditions [33,34,35]. Moreover, the time of reaction was dramatically reduced from 12 h (720 min) in the conventional synthesis (Method B) to 10 min for the MWAS method. The presence of two distinct alkoxy groups in the three positions of the starting dihydropyridine derivative **I** did not significantly alter the yields obtained.

**Scheme 5:**
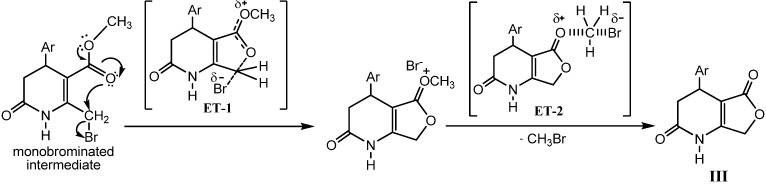
The mechanism and the transition states postulated for the second step of the lactonization reaction.

Lactonization could be accounted for by the Wohl-Ziegler bromination (allylic bromination) [36] at the methyl group to the 2nd position of the heterocycle **I**, yielding the non-isolable monobrominated intermediate via a free radical process, followed by intramolecular cyclization to give the corresponding γ-lactone (Scheme 5) in similar way to the pyridinium bromide perbromide procedure reported for 1,4-DHPs [37]. Given the higher reactivity of the radical species, we propose that the second stage is the determining step in the mechanism postulated for this reaction. The intramolecular cyclization could take place through a polar mechanism. The non-isolable monobrominated intermediate leads to a charged species as a result of an intramolecular nucleophilic attack through SN2 mechanism. Subsequently, the bromide originated in this process could act as a nucleophile on the methyl carbon, with the loss of a bromomethane molecule, to obtain the final product of reaction **III** (Scheme 5). In both postulated steps for this second stage, the polarity is increased from the ground states to the transition states (**ET-1** and **ET-2**) (Scheme 5), thus resulting in an enhancement of reactivity under MW irradiation by lowering the activation energy [30].

It is possible that the increase in the rate of the second stage of the lactonization reaction under MW irradiation prevents the accumulation of the monobrominated intermediate, thus reducing the risk of polybromation and consequently increasing yields.

The final products **IIIa–i** were characterized by melting point, NMR and mass spectral data. Most compounds synthesized in this study were known and their spectral characterization showed satisfactory agreement with the previous literature data [33,34,35]. The ^1^H-NMR spectra of DHP derivatives **IIIa–i** showed one singlet corresponding to the NH at δ ~ 10.7–11.3 ppm. The signals corresponding to the lactone ring methylene protons appeared as an AB system at δ 4.97 and δ 5.37 ppm, due to the germinal coupling between them, and confirmed the formation of lactone-fused DHP. The ^13^C-NMR spectra of these compounds displayed signals in the carbonyl, aromatic, and aliphatic regions. For the nitrogen heterocyclic ring, the spectra showed three quaternary carbon signals (C-2, C-4a, and C-7a), one secondary carbon signal (C-4), and one primary carbon signal (C-3). The signals of the quaternary carbons C-7a appeared at higher δ values than those expected for typical olefinic carbon atoms. In contrast, the quaternary carbon C-4a was observed at unusually lower δ values. This displacement of the signals is due to the strong push–pull effect of the groups linked to the olefinic double bonds [33,34,35].

The MWAS without solvent of furopyridone derivatives **IIIa–i** (Method A) thus offers considerable advantages over our previous reported conventional lactonization synthesis [33,34,35]. The reaction time was notably reduced [conventional synthesis (Method B): 12 h, and MWAS (Method A): 10 min), and the final product was obtained in excellent purity and yield and hence could be used in further procedures without the need for any wasteful purification steps.

## 3. Experimental

### 3.1. General

Reagents and solvents were purchased from Fluka or Aldrich. The progress of the reaction and the purity of compounds were monitored on TLC analytical silica gel plates (Merck 60F250) using *n*-hexane-chloroform-ethyl acetate (3:2:1) and benzene-methanol (7:2) as eluents for the compounds **IIa–j** and **IIIa–I**, respectively. The MW irradiation was provided by a CEM Discover LabMate Focused Single Mode MW Synthesis Reactor, which produced continuous stirring and irradiation with control of pressure and temperature. Melting points were determined in capillary tubes in an Electrothermal C14500 apparatus and are uncorrected. The NMR spectra were recorded on a Mercury 400 spectrometer [400 MHz (^1^H) and 75.4 MHz (^13^C)]. Chemical shifts are given as δ values against tetramethylsilane as the internal standard and *J* values are given in Hz. Mass spectra were obtained in a LC/MSD-TOF(2006) Instrument (Agilent Technologies).

### 3.2. General Procedure for the MWAS of 4-Arylsubstituted alkyl 1,4,5,6-Tetrahydro-2-methyl-6-oxopyridine-3-carboxylates ***I***

4-Arylsubstituted alkyl 1,4,5,6-tetrahydro-2-methyl-6-oxopyridine-3-carboxylates **I** were prepared following the previously reported procedure [20]. In this case the MW irradiation was provided by a CEM Discover LabMate Focused Single Mode MW Synthesis Reactor, and the reaction mixtures were irradiated for 10 min at 250 Watts. All the compounds were characterized by determination of physical constants and by NMR spectroscopy, which coincided with those previously reported for these compounds [20].

### 3.3. General Procedure for the MWAS of Methyl 4-Arylsubstituted-6-chloro-5-formyl-2-methyl-1,4-dihydropyridine-3-carboxylates ***IIa–j***

4-Aryl-substituted alkyl 1,4,5,6-tetrahydro-2-methyl-6-oxopyridine-3-carboxylates (**I**, 7 mmol) were added to the Vilsmeier-Haack reagent prepared from a mixture of POCl_3_ (1.1 mL, 12.2 mmol) and DMF (1.4 mL, 18.2 mmol) at 5 °C. This mixture was then irradiated in the CEM Discover reactor at 180 Watts for 5 min at the controlled temperature of 50 °C. After the completion of the reaction, an aqueous sodium acetate solution was added (12 g in 21 mL of water). After 0.5 h, the mixture was partitioned between water and chloroform, and the aqueous phase was extracted with ethyl acetate. The organic phases were mixed and dried with anhydrous magnesium sulfate. The organic solvent was removed *invacuo* and the solid was precipitated from diethyl ether, filtered and washed with small portions of cooled ethanol. The chracterization data of the compounds is given below.

*Methyl 6-chloro-5-formy1-2-methyl-4-(2′-nitrophenyl)-1,4-dihydropyridine-3-carboxylate* (**IIa**). Yellow solid; m.p. 178–180 °C; yield: 69%; ^1^H-NMR (DMSO-d_6_) δ 2.34 (s, 3H, CH_3_), 3.55 (s, 3H, OCH_3_), 4.95 (s, 1H, H4), 7.64–7.53 (m, 3H, Ar), 8.04 (dt, *J* = 7.7 Hz, *J* = 2.1 Hz, 1H, Ar), 9.65 (s, 1H, CHO), 10.56 (s, 1H, NH); ^13^C-NMR (DMSO-d_6_) δ 18.6 (CH_3_), 39.0 (C4), 60.6 (OCH_3_), 111.3 (C3), 104.8 (C5), 122.3 (C5′), 130.1 (C3′), 134.0 (C4′), 135.0 (C6′), 144.1 (C2), 147.1 (C6), 147.3 (C2′), 148.6 (C1′), 166.4 (COOCH_3_), 187.3 (CHO); ESI-MS: *m/z* 337 [M+H]^+^.

*Methyl 6-chloro-5-formy1-2-methyl-4-(3′-nitrophenyl)-1,4-dihydropyridine-3-carboxylate* (**IIb**). Yellow solid; m.p. 213–214 °C; yield: 68%; ^1^H-NMR (DMSO-d_6_) δ 2.36 (s, 3H, CH_3_), 3.54 (s, 3H, OCH_3_), 5.05 (s, 1H, H4), 7.62–7.58 (m, 2H, Ar), 8.00 (t, *J* = 2.0 Hz, 1H, Ar), 8.05 (dt, *J* = 7.5 Hz, *J* = 2.0 Hz, 1H, Ar), 9.67 (s, 1H, CHO), 10.53 (s, 1H, NH); ^13^C-NMR (DMSO-d_6_) δ 17.9 (CH_3_), 37.9 (C4), 51.2 (OCH_3_), 103.6 (C5), 110.4 (C3), 120.8 (C2′), 121.0 (C4), 129.6 (C6′), 133.6 (C5), 143.5 (C2), 147.6 (C3′), 147.7 (C6), 149.3 (C1′), 166.2 (COOCH_3_), 186.5 (CHO); ESI-MS: *m/z* 337 [M+H]^+^.

*Methyl 6-chloro-5-formyl-2-methyl-4-(4′-nitrophenyl)-1,4-dihydropyridine-3-carboxylate* (**IIc**). Yellow solid; m.p. 190–192 °C; yield: 65%; ^1^H-NMR (DMSO-d_6_) δ 2.34 (s, 3H, CH_3_), 3,54 (s, 3H, OCH_3_), 5.03 (s, 1H, H4), 7.44 (d, *J* = 7.9 Hz, 2H, Ar), 8,12 (d, *J* = 7.9 Hz, 2H, Ar), 9,67 (s, 1H, CHO), 10.48 (s, 1H, NH); ^13^C-NMR (DMSO-d_6_) δ ppm 18.7 (CH_3_), 39.2 (C4), 51.2 (OCH_3_), 104.6 (C5), 111.1 (C3), 124.4 (C3′,C5′), 129.6 (C2′,C6′), 141.1 (C2), 147.1 (C4′), 146.9 (C6), 153.7 (C1′), 166.6 (COOCH_3_), 187.4 (CHO); ESI-MS: *m/z* 337 [M+H]^+^.

*Methyl 6-chloro-5-formyl-4-(4-methoxycarbonylphenyl)-2-methyl-l,4dihydropyridine-3-carboxylate* (**IId**). White solid; mp 202–204 °C; yield: 63%; ^1^H-NMR (DMSO-d_6_) δ 2.34 (s, 3H, CH_3_), 3.53 (s, 3H, OCH_3_), 3.79 (s, 3H, OCH_3_), 5.00 (s, 1H, CH), 7.29 (d, *J* = 7.9 Hz, 2H, Ar), 7.84 (d, *J* = 7.9 Hz, 2H, Ar), 9.67 (s, 1H, HCO), 10.46 (s, 1H, NH); ^13^C-NMR (DMSO-d_6_) δ 17.8 (CH_3_), 38.0 (C4), 51.1 (OCH_3_), 103.8 (C3), 110.5 (C5), 127.6 (C3′,C5′), 129.3 (C2′,C6′), 143.1 (C2),146.1 (C6), 150.7 (C1′), 166.4 (COOCH_3_), 186.5 (CHO); ESI-MS: *m/z* 350 [M+H]^+^.

*Methyl 4-(2′,3′-dihidroxyphenyl)-6-chloro-5-formyl-2-methyl-l,4dihydropyridine-3-carboxylate* (**IIe**). White solid; mp 239–241 °C; yield: 70%; ^1^H-NMR (DMSO-d_6_) δ 2.24 (s, 3H, CH_3_), 3,77 (s, 3H, OCH_3_), 4.98 (s, 1H, H4), 5.40 (brs, 2H, OH), 6.62(m, 3H, Ar) 9.71 (s, 1H, HCO), 10.38 (s, 1H, NH); ^13^C-NMR (DMSO-d_6_) δ 17.8 (CH_3_), 38.2 (C4), 51.3 (OCH_3_), 103.7 (C3), 110.3 (C5), 114.3 (C4′), 122.2 (C5′), 123.2 (C6′), 124.1 (C1′), 145.2 (C3′), 147 (C2′), 143.1 (C2), 146.5 (C6), 150.3 (C1′), 166.6 (COOCH_3_), 186.8 (CHO); ESI-MS: *m/z* 324 [M+H]^+^.

*Methyl 6-chloro-4-(4′-dimethylaminophenyl)-5-formyl-2-methyl-1,4-dihydropyridine-3-carboxylate* (**IIf**). Yellow solid; m.p. 290–292 °C; yield: 60%; ^1^H-NMR (DMSO-d_6_) δ 2.34 (s, 3H, CH_3_), 3.10 (s, 6H, CH_3_), 3,52 (s, 3H, OCH_3_), 5.00 (s, 1H, H4), 7.39 (d, *J* = 7.9 Hz, 2H, Ar), 8,08 (d, *J* = 7.9 Hz, 2H, Ar), 9,77 (s, 1H, CHO), 10.48 (s, 1H, NH); ^13^C-NMR (DMSO-d_6_) δ 18.7 (CH_3_), 39.2 (C4), 41.7 (2CH_3_), 51.2 (OCH_3_), 104.4 (C5), 110.9 (C3), 124.2 (C3′,C5′), 129.8 (C2′,C6′), 141.1 (C2), 147.2 (C4′), 146.7 (C6), 153.5 (C1′), 166.8 (COOCH_3_), 187.2 (CHO). ESI-MS: *m/z* 335 [M+H]^+^.

*Ethyl 6-chloro-5-formyl-2-methyl-4-phenyl-1,4-dihydropyridine-3-carboxylate* (**IIg**). White solid; m.p. 201–202 °C; yield: 62%; ^1^H-NMR (DMSO-d_6_) δ 1.10 (t, *J* = 7.1 Hz, 3H, CH_3_), 2.33 (s, 3H, CH_3_), 4.02 (q, *J* = 7.1 Hz, 2H, OCH_2_), 4.93 (s, 1H, H4), 7.09 (d, *J* = 7.8 Hz, 2H, Ar), 7.31–7.11 (m, 3H, Ar), 9.72 (s, 1H, CHO), 10.35 (s, 1H, NH); ^13^C-NMR (DMSO-d_6_) δ 14.6 (CH_3_), 18.3 (CH_3_), 38.1 (C4), 59.8 (OCH_2_), 103.9 (C5), 109.3 (C3), 126.1 (C4′), 126.6 (C2′,C6′), 128.2 (C3′,C5′), 144.3 (C2), 146.9 (C1′), 147.9 (C6), 166.2 (COOCH_2_CH_3_), 187.4 (CHO); ESI-MS: *m/z* 306 [M+H]^+^. 

*Ethyl 6-chloro-5-formyl-2-methyl-4-(2′-nitrophenyl)-1,4-dihydropyridine-3-carboxylate* (**IIh**). Yelow solid; m.p. 198–200 °C; yield: 65%; ^1^H-NMR (DMSO-d_6_) δ 1.10 (t, *J* =7.1 Hz, 3H, CH_3_), 2.35 (s, 3H, CH_3_), 3.99 (q, *J* =7.1 Hz, 2H, OCH_2_), 5.03 (s, 1H, H4), 7.64–7.53 (s, 2H, Ar), 7.96 (t, *J* = 2.0 Hz, 1H, Ar), 8.02 (dt, *J* = 7.7 Hz, *J* = 2.0 Hz, 1H, Ar), 9.68 (s, 1H, CHO), 10.52 (1H, s, NH); ^13^C-NMR (DMSO-d_6_) δ 14.8 (CH_3_), 18.6 (CH_3_), 39.0 (C4), 60.6 (OCH2), 104.8 (C5), 111.3 (C3), 122.4 (C3′), 122.7 (C5′), 130.7 (C4′), 135.0 (C6′), 144.1 (C2), 147.1 (C6), 148.4 (C2′), 148,6 (C1′), 166.5 (COOCH2CH3), 187.4 (CHO); ESI-MS: *m/z* 351 [M+H]^+^.

*Ethyl 4-(2′,3′-dihidroxyphenyl)-6-chloro-5-formyl-2-methyl-l,4dihydropyridine-3-carboxylate* (**IIi**). White solid; mp 248–250 °C; yield: 63%; ^1^H-NMR (DMSO-d_6_) δ 1.28 (t, 3H, CH_3_), 4,17 (q, 2H, OCH_2_), 4.98 (s, 1H, H4), 5.40 (brs, 2H, OH), 6.62(m, 3H, Ar) 9.71 (s, 1H, HCO), 10.38 (s, 1H, NH); ^13^C-NMR (DMSO-d_6_) δ 14.8 (CH_3_), 18.5 (CH_3_), 38.2 (C4), 61.3 (OCH_2_), 103.7 (C3), 110.3 (C5), 114.3 (C4′), 122.2 (C5′), 123.2 (C6′), 124.1 (C1′), 145.2 (C3′), 147 (C2′), 143.1 (C2),146.5 (C6), 150.3 (C1′), 166.6 (COOCH_3_), 186.8 (CHO); ESI-MS: *m/z* 338 [M+H]^+^.

*Ethyl 6-chloro-4-(4′-dimethylaminophenyl)-5-formyl-2-methyl-1,4-dihydropyridine-3-carboxylat*e (**IIj**). Yellow solid; m.p. 300–302 °C; yield: 64%; ^1^H-NMR (DMSO-d_6_) δ 1.25 (t, 3H, CH_3_), 3.10 (s, 6H, CH_3_), 4,17 (q, 2H, OCH_2_), 4.89 (s, 1H, H4), 7.35 (d, *J* = 7.9 Hz, 2H, Ar), 8,02 (d, *J* = 7.9 Hz, 2H, Ar), 9,87 (s, 1H, CHO), 10.48 (s, 1H, NH); ^13^C-NMR (DMSO-d_6_) δ 14.5 (CH_3_), 18.3 (CH_3_), 39.2 (C4), 41.7 (2CH_3_), 61.5 (OCH_2_), 104.4 (C5), 110.9 (C3), 124.2 (C3′,C5′), 129.8 (C2′,C6′), 141.1 (C2), 147.2 (C4′), 146.7 (C6), 153.5 (C1′), 166.8 (COOCH_3_), 187.2 (CHO). ESI-MS: *m/z* 349 [M+H]^+^.

### 3.4. General Procedure for the MWAS of 4-Arylsubstituted-4,7-dihydrofuro[3,4-b]pyridine-2,5(1H,3H)-diones ***IIIa–i***

A mixture of 4-aryl-substituted alkyl 1,4,5,6-tetrahydro-2-methyl-6-oxopyridine-3-carboxylate (**I**, 5 mmol) and *N*-bromosuccinimide (0.89 g, 5 mmol) was irradiated without solvent in the CEM Discover reactor at 240 Watts for 10 min, and at the controlled temperature of 80 °C. When the irradiation was stopped, the mixture was treated with chloroform, and the solid obtained was filtered and washed with small portion of cool chloroform and diethyl ether to give the pure products. Compound data is given below.

*4-(3′-Nitrophenyl)-3,4-dihydrofuro[3,4-b]pyridine-2,5-(1H,7H)-dione* (**IIIa**). Pale yellow solid; m.p. 218–219 °C; yield: 80% from I (R = Me), and 85% from I (R = Et); ^1^H-NMR (DMSO-d_6_) δ 3.03 (dd, 1H, H3b, *J* = 16.7 Hz, *J* = 3.8 Hz B part of ABX), 3.64 (dd, 1H, H3a, *J* = 16.7 Hz, *J* = 8.9 Hz A part of ABX), 4.85 (dd, 1H, H4, *J* = 8.9 Hz, *J* = 3.8 Hz, X part of ABX), 5.34 (dd, 2H, OCH_2_), 8.38–7.82 (m, 4H, Ar), 11.27 (s, 1H, NH); ^13^C-NMR (DMSO-d_6_) δ 29.6 (C4), 38.1 (C3), 65.5 (C7), 99.8 (C4a), 124.7 (C5′), 128.7 (C4′), 129.1 (C6′), 133.8 (C3′), 135.1 (C1′), 148.6 (C2′), 162.0 (C7a), 168.0 (C5), 170.4 (C2); ESI-MS: *m/z* 275 [M+H]^+^.

*4-(3′-Nitrophenyl)-3,4-dihydrofuro[3,4-b]pyridine-2,5-(1H,7H)-dione* (**IIIb**). Pale yellow solid; m.p. 234–235 °C; yield: 79% from I (R = Me), and 82% from I (R = Et); ^1^H-NMR (DMSO-d_6_) δ 2.67 (d, 1H, H3b, *J* = 16.9 Hz, *J* = 8.9 Hz B part of ABX) 3.15 (dd, 1H, H3a, *J* = 16.6 Hz, *J* = 8.9 Hz A part of ABX), 4 .25 (dd, 1H, H4, *J* = 9.0 Hz, *J* = 3.6 Hz X part of ABX); 4.97 (dd, 2H, OCH_2_), 8.12–7.64 (m, 4H, Ar), 10.86 (s, 1H, NH); ^13^C-NMR (75 MHz, DMSO-d_6_) δ 33.1 (C4), 38.0 (C3), 65.5 (C7), 100.5 (C4a), 148.0; 143.9; 133.6; 130.3; 122.1; 121.6 (C aromatics), 161.4 (C7a), 169.3 (C5), 170.8 (C2); ESI-MS: *m/z* 275 [M+H]^+^.

*4-(4′-Nitrophenyl)-3,4-dihydrofuro[3,4-b]pyridine-2,5-(1H,7H)-dione* (**IIIc**). Pale yellow solid; m.p. 241–243 °C; yield: 82% from I (R = Me), and 85% from I (R = Et); ^1^H-NMR (DMSO-d_6_) δ 2.67 (dd, 1H, H3b, *J* = 16.6 Hz, *J* = 3.6 Hz B part of ABX), 3.15 (dd, 1H, H3a, *J* = 16.6 Hz, *J* = 8.9 Hz A part of ABX), 4.25 (dd, 1H, H4, *J* = 9.0 Hz, *J* = 3.6 Hz, X part of ABX), 4.97 (dd, 2H, OCH_2_), 8.15–7.42 (m; 4H; Ar), 10.81 (s, 1H, NH); ^13^C-NMR (DMSO-d_6_) δ 33.1 (C4), 38.0 (C3), 65.5 (C7), 100.5 (C4a), 148.0, 143.9, 133.6, 130.3, 122.1,121.6 (C aromatics), 161.4 (C7a), 169.3 (C5), 170.8 (C2); ESI-MS: *m/z* 275 [M+H]^+^.

*4-(4′-Tolyl)-3,4-dihydrofuro[3,4-b]pyridine-2,5-(1H,7H)-dione* (**IIId**). White solid; m.p. 200–202 °C; yield: 89% from I (R = Me), and 85% from I (R = Et); ^1^H-NMR (DMSO-d_6_) δ 2.65 (dd, 1H, H3b, *J* = 16.6 Hz, *J* = 3.6 Hz B part of ABX), 3.15 (dd, 1H, H3a, *J* = 16.6 Hz, *J* = 8.9 Hz A part of ABX), 4.26 (dd, 1H, H4, *J* = 9.0 Hz, *J* = 3.6 Hz, X part of ABX), 4.95 (dd, 2H, OCH_2_), 7.32–8.12 (m; 4H; Ar), 10.78 (s, 1H, NH); ^13^C-NMR (DMSO-d_6_) δ 33.3 (C4), 38.2 (C3), 65.3 (C7), 100.4 (C4a), 121.5, 122.5, 130.1, 133.6, 144.0, 148.1 (C aromatics), 161.4 (C7a), 169.3 (C5), 170.8 (C2); ESI-MS: *m/z* 244 [M+H]^+^.

*4-(2′,3′-Dihydroxyphenyl)-3,4-dihydrofuro[3,4-b]pyridine-2,5-(1H,7H)-dione* (**IIIe**). White solid; m.p. 291–293 °C; yield: 72% from I (R = Me), and 79% from I (R = Et); ^1^H-NMR (DMSO-d_6_) δ 2.65 (d, 1H, H3b, *J* = 16.9 Hz, *J* = 8.9 Hz B part of ABX) 3.10 (dd, 1H, H3a, *J* = 16.6 Hz, *J* = 8.9 Hz A part of ABX), 4.18 (dd, 1H, H4, *J* = 9.0 Hz, *J* = 3.6 Hz X part of ABX); 4.92 (dd, 2H, OCH_2_), 7.54–8.08 (m, 3H, Ar), 10.88 (s, 1H, NH); ^13^C-NMR (DMSO-d_6_) δ 30.9 (C4), 38.5 (C3), 65.3 (C7), 99.8 (C4a), 145.2; 145.9; 130.6; 123.7; 122.8; 119.9 (C aromatics), 162.1 (C7a), 169.3 (C5), 170.5 (C2); ESI-MS: *m/z* 262 [M+H]^+^.

*4-(4′-(Dimethylamino)phenyl)-3,4-dihydrofuro[3,4-b]pyridine-2,5-(1H,7H)-dione* (**IIIf**). Yellow solid; m.p. 243–245 °C; yield: 84% from I (R = Me), and 85% from I (R = Et); ^1^H-NMR (DMSO-d_6_) δ 2.63 (d, 1H, H3b, *J* = 16.9 Hz, *J* = 8.9 Hz B part of ABX), 2.99 (s, 6H, 2CH_3_), 3.05 (dd, 1H, H3a, *J* = 16.6 Hz, *J* = 8.9 Hz A part of ABX), 4.09 (dd, 1H, H4, *J* = 9.0 Hz, *J* = 3.6 Hz X part of ABX); 4.85 (dd, 2H, OCH_2_), 7.14–7.78 (m, 4H, Ar), 10.88 (s, 1H, NH); ^13^C-NMR (DMSO-d_6_) δ 32.8 (C4), 38.5 (C3), 40.9 (NCH_3_), 65.2 (C7), 99.9 (C4a), 119.9, 120.2; 128.9; 129.6, 132.7; 148.2, (C aromatics), 162.0 (C7a), 169.0 (C5), 170.1 (C2); ESI-MS: *m/z* 273 [M+H]^+^.

*4-Phenyl-3,4-dihydrofuro[3,4-b]pyridine-2,5-(1H,7H)-dione* (**IIIg**). White solid; m.p. 239–240 °C; yield: 84% from I (R = Me), and 85% from I (R = Et); ^1^H-NMR (DMSO-d_6_) δ 2.57 (d, 1H, H3b, *J* = 16.6 Hz, *J* = 3.6 Hz B part of ABX), 3.12 (dd, 1H, H3a, *J* = 16.6 Hz, *J* = 8.9 Hz A part of ABX), 4.01 (d, 1H, H4, *J* = 9.0 Hz, *J* = 3.6 Hz, X part of ABX), 4.91 (dd, 2H, OCH_2_), 7.18–7.35 (m; 4H; Ar), 10.74 (s, 1H, NH); ^13^C-NMR (DMSO-d_6_) δ 33.3 (C4), 38.4 (C3), 65.3 (C7), 101.7 (C4a), 126.5, 126.9, 128.7, 144.8 (C aromatics), 160.7 (C7a), 169.0 (C5), 170.9 (C2); ESI-MS: *m/z* 230 [M+H]^+^.

*4-(3′-Methoxyphenyl)-3,4-dihydrofuro[3,4-b]pyridine-2,5-(1H,7H)-dione* (**IIIh**). Pale yellow solid; m.p. 268–270 °C; yield: 88% from I (R = Me), and 85% from I (R = Et); ^1^H-NMR (DMSO-d_6_) δ 2.61 (d, 1H, H3b, *J*=16.9 Hz, *J*= 8.9 Hz B part of ABX) 3.14 (dd, 1H, H3a, *J* = 16.6 Hz, *J* = 8.9 Hz A part of ABX), 3.75 (s, 3H, OCH_3_), 4.20 (dd, 1H, H4, *J* = 9.0 Hz, *J* = 3.6 Hz X part of ABX); 4.82 (dd, 2H, OCH_2_), 7.43–8.12 (m, 4H, Ar), 10.70 (s, 1H, NH); ^13^C-NMR (DMSO-d_6_) δ 33.5 (C4), 38.3 (C3), 53.2 (OCH_3_), 65.3 (C7), 101.0 (C4a), 120.9, 121.9, 130.3, 133.4, 141.9, 158.2 (C aromatics), 161.8 (C7a), 169.0 (C5), 170.1 (C2), ESI-MS: *m/z* 260 [M+H]^+^.

*4-(3′,4′,5′-Trimethoxyphenyl)-3,4-dihydrofuro[3,4-b]pyridine-2,5-(1H,7H)-dione* (**IIIi**). Yellow solid; m.p. 288–290 °C; yield: 83% from I (R = Me), and 81% from I (R = Et); ^1^H-NMR (DMSO-d_6_) δ 2.63 (d, 1H, H3b, *J* = 16.9 Hz, *J* = 8.9 Hz B part of ABX) 3.10 (dd, 1H, H3a, *J* = 16.6 Hz, *J* = 8.9 Hz A part of ABX), 3.83 (s, 9H, 3OCH_3_), 4.18 (dd, 1H, H4, *J* = 9.0 Hz, *J* = 3.6 Hz X part of ABX); 4.82 (dd, 2H, OCH_2_), 7.53 (s, 2H, Ar), 10.70 (s, 1H, NH); ^13^C-NMR (DMSO-d_6_) δ 33.5 (C4), 38.3 (C3), 56.2 (2OCH_3_), 60.8 (OCH_3_), 65.3 (C7), 101.0 (C4a), 109.9, 134.9, 136.5, 153.4 (C aromatics), 161.9 (C7a), 169.2 (C5), 170.3 (C2), ESI-MS: *m/z* 320 [M+H]^+^.

## 4. Conclusions

The MW-assisted methods presented here for the synthesis of alkyl 4-arylsubstituted-6-chloro-5-formyl-2-methyl-1,4-dihydropyridine-3-carboxylates **IIa–j** and 4-arylsubstituted-4,7-dihydrofuro[3,4-*b*]pyridine-2,5-(1*H*,3*H*)-diones **IIIa–i** are straightforward, mild, and efficient. In both cases, the overall process was more energy-efficient than classical heating, since direct “in-core” heating of the medium occurred. These protocols have advantages over other techniques as they offer a shorter reaction times, cleaner reaction profiles, solvent-free reactions (for compounds **IIIa–i**), higher yields and an easy eco-friendly work-up.
